# Joint Modeling of Social Determinants and Clinical Factors to Define Subphenotypes in Out-of-Hospital Cardiac Arrest Survival: Cluster Analysis

**DOI:** 10.2196/51844

**Published:** 2023-12-06

**Authors:** Ethan E Abbott, Wonsuk Oh, Yang Dai, Cole Feuer, Lili Chan, Brendan G Carr, Girish N Nadkarni

**Affiliations:** 1 Department of Emergency Medicine, Icahn School of Medicine at Mount Sinai, New York, NY, United States; 2 Institute for Health Equity Research, Icahn School of Medicine at Mount Sinai, New York, NY, United States; 3 Division of Data-Driven and Digital Medicine, Icahn School of Medicine at Mount Sinai, New York, NY, United States; 4 Department of Population Health Science and Policy, Icahn School of Medicine at Mount Sinai, New York, NY, United States; 5 Department of Genetics and Genomic Sciences, Icahn School of Medicine at Mount Sinai, New York, NY, United States; 6 The Charles Bronfman Institute for Personalized Medicine, Icahn School of Medicine at Mount Sinai, New York, NY, United States; 7 Division of Nephrology, Department of Medicine, Icahn School of Medicine at Mount Sinai, New York, NY, United States

**Keywords:** out-of-hospital-cardiac arrest, machine learning, social determinants of health, SDOH, cluster, cardiac, heart, cardiology, myocardial, phenotype, phenotypes, subphenotype, subphenotypes, mortality, death, survive, survival, survivor, survivors, retrospective, observational, cohort, algorithm, algorithms, k-means, clustering, association, associations

## Abstract

**Background:**

Machine learning clustering offers an unbiased approach to better understand the interactions of complex social and clinical variables via integrative subphenotypes, an approach not studied in out-of-hospital cardiac arrest (OHCA).

**Objective:**

We conducted a cluster analysis for a cohort of OHCA survivors to examine the association of clinical and social factors for mortality at 1 year.

**Methods:**

We used a retrospective observational OHCA cohort identified from Medicare claims data, including area-level social determinants of health (SDOH) features and hospital-level data sets. We applied k-means clustering algorithms to identify subphenotypes of beneficiaries who had survived an OHCA and examined associations of outcomes by subphenotype.

**Results:**

We identified 27,028 unique beneficiaries who survived to discharge after OHCA. We derived 4 distinct subphenotypes. Subphenotype 1 included a distribution of more urban, female, and Black beneficiaries with the least robust area-level SDOH measures and the highest 1-year mortality (2375/4417, 53.8%). Subphenotype 2 was characterized by a greater distribution of male, White beneficiaries and had the strongest zip code–level SDOH measures, with 1-year mortality at 49.9% (4577/9165). Subphenotype 3 had the highest rates of cardiac catheterization at 34.7% (1342/3866) and the greatest distribution with a driving distance to the index OHCA hospital from their primary residence >16.1 km at 85.4% (8179/9580); more were also discharged to a skilled nursing facility after index hospitalization. Subphenotype 4 had moderate median household income at US $51,659.50 (IQR US $41,295 to $67,081) and moderate to high median unemployment at 5.5% (IQR 4.2%-7.1%), with the lowest 1-year mortality (1207/3866, 31.2%). Joint modeling of these features demonstrated an increased hazard of death for subphenotypes 1 to 3 but not for subphenotype 4 when compared to reference.

**Conclusions:**

We identified 4 distinct subphenotypes with differences in outcomes by clinical and area-level SDOH features for OHCA. Further work is needed to determine if individual or other SDOH domains are specifically tied to long-term survival after OHCA.

## Introduction

More than 400,000 incidents of out-of-hospital cardiac arrest (OHCA) occur each year in the United States, with low rates of survival [1-4]. Despite poor outcomes, there have been noted improvements in rates of survival over the last decade, leading to a renewed focus on postdischarge longitudinal trajectories. Drivers of disparities in long-term outcomes after OHCA are not well understood and are potentially affected by nonclinical factors. Social determinants of health (SDOH) represent key social, living, and environmental conditions where people reside and work [5]. SDOH are linked to racial, ethnic, and socioeconomic disparities in health outcomes for multiple chronic health conditions [6-9]. Several of these SDOH domains are noted to be important factors in short-term OHCA survival, but the relationships with longer-term outcomes and area-level SDOH have not been deeply explored [10-12]. While individual-level SDOH information provides granular patient-level information, screening and collection of this data can be resource intensive and has been inconsistently collected by health systems and organizations [13]. Area-level SDOH data derived from the US Census at the neighborhood, census tract, zip code, or regional level is highly accessible; linkages to existing health care data sets can provide insight into the association of key social and living environments with clinical outcomes.

Unsupervised machine learning cluster analysis is a methodologic approach that seeks to discover hidden patterns in unlabeled data and can be used to identify distinct subgroups of patients that share certain characteristics that can be tied to specific clinical end points. The primary objective of this approach is to group observations that share similarities in their features or characteristics, allowing the identification of distinct subgroups of patients with similar traits. These subgroups can then be correlated with specific clinical end points, providing valuable insights into disease pathogenesis and potential therapeutic targets. This can function to further elucidate specific clinical subphenotypes of patients and better understand the interactions of complex variables. Prior clustering methods have successfully identified subphenotypes of COVID-19 patients in the intensive care unit, disparities in Black kidney transplant recipients’ outcomes, and clusters of patients with high mortality in sepsis [14-16]. Differences in outcomes might be better captured through clustering methodology that could reveal similarities or differences in subgroups of patients to better understand this interaction between SDOH factors and health outcomes. By jointly modeling features, a comprehensive model can account for multiple data sources or features, improving performance over separate models.

The complex interaction between community-level SDOH and clinical factors has undergone limited study in OHCA, and prior work examining outcomes for Medicare beneficiaries has not been explored deeply. Because of this, we undertook an analysis of a Medicare OHCA cohort who survived to discharge using unsupervised machine learning clustering approaches to examine if clinical, demographic, and important SDOH domains are associated with differences in mortality at 1 year.

## Methods

### Study Population

For this analysis, we used a retrospective observational cohort of age-eligible (≥65 years) Medicare fee-for-service claims data from the Medicare Provider Analysis and Review (MedPAR) and outpatient research identifiable files (RIFs) for January 2013 through December 2015. We identified individual patient demographics, including race and ethnicity, sex, and age from the Medicare Beneficiary Summary file. We included beneficiaries with emergency department (ED)–treated OHCA using claims with *International Classification of Diseases, Ninth Revision, Clinical Modification* (*ICD-9-CM*) codes 427.5, 427.4, 427.41, and 427.42; we mapped *ICD-10-CM* codes I46, I49.0, I49.01, and I49.02 as the primary or admitting diagnosis based on prior approaches used for identifying OHCA patients [17-20]. Beneficiary zip code–level residence was determined from the primary claim present at index admission for OHCA.

### Features of Interest

We included a total of 28 continuous and categorical features that were incorporated into our models (Table S1 in Multimedia Appendix 1). These features were selected based on prior OHCA research conducted using Centers for Medicare and Medicaid Services (CMS) data [21] and variables closely aligned with OHCA outcomes. Medicare beneficiary demographics were abstracted from the index hospital claim: age category, sex, and race and ethnicity. Race and ethnicity were classified as Black, White, or “other.” The “other” category included CMS-defined racial and ethnic groups: Hispanic; Asian, Native Hawaiian, or Pacific Islander; and American Indian or Alaska Native. Beneficiary comorbidities were identified from the MedPAR or outpatient RIFs and summarized using the Agency for Healthcare Research and Quality Elixhauser Comorbidity Index that were present on admission [22,23]. We also identified beneficiaries who underwent cardiac catheterization and implantable cardioverter defibrillator placement at the index hospitalization for OHCA using documented procedure codes. We determined beneficiaries who underwent interhospital transfer at index hospitalization and those with a prior claim at a skilled nursing facility (SNF) or inpatient stay prior to index OHCA hospitalization. We calculated total length of stay (LOS) for each beneficiary and if they were discharged to a SNF after index hospitalization for OHCA.

For hospital-level variables*,* we selected key hospital-level characteristics from the American Hospital Association Survey data set that could impact care. Hospital characteristics included total number of hospital beds and hospital teaching status (major academic teaching, minor academic teaching). We also estimated the driving distance to the nearest hospital for each beneficiary based on primary zip code–level residence (<8.0 km, 8.0 km-16.1 km, >16.1 km). The driving distance was calculated using the Open-Source Routing Machine (OSRM) library [24].

We used the US Census Bureau American Community Survey 5-year estimates to identify key zip code–level SDOH domains. These domains were selected based on expert consensus and from prior research using claims data [25]. We mapped selected SDOH features to the residential zip code documented on the index OHCA claim. For SDOH features, we included the following at the zip code level: (1) median household income (HHI), (2) percentage unemployed, (3) percentage below the poverty line, (3) percentage with a high school education or higher, (4) percentage with a bachelor’s degree or higher, and (4) percentage who drive alone. To characterize urban-rural status, the 2013 National Center for Health Statistics (NCHS) urban-rural classification was used, using the residential zip code identified on the first claim of each encounter. We classified urban-rural status into three categories: (1) large metropolitan urban, (2) small/midmetropolitan, and (3) nonmetropolitan.

### Study Outcomes

Our primary outcome was mortality at 1 year from index OHCA. Beneficiary date of death was determined from the Vital Status File, including validated dates of death up to June 2019.

### Data Processing and Subphenotype Development

We applied several preprocessing steps to our data set to address outliers, including 95% Winsorization and log transformation of features with skewed distributions, using a total of 28 features for clustering analysis. We selected Winsorization over other approaches given extreme values within the SDOH data set. Beneficiaries that had any missing features of interest were excluded from the final analytic data set.

### Cluster Analysis

We used k-means clustering to extract subphenotypes. The final optimal number of clusters was determined from the results generated from the NbClust package in R (version 4.04; R Foundation for Statistical Computing) [26]. We evaluated the robustness of the subphenotypes by rederiving them from hierarchical clustering, assessing the consistency of the subphenotypes from both the k-means and hierarchical approaches visually on uniform manifold approximation and projection (UMAP) spaces (Figure S1 in Multimedia Appendix 2 and Figure S2 in Multimedia Appendix 3). We also numerically examined the agreement of the subphenotype membership using Sankey diagrams and multiclass area under the receiver operating characteristic (ROC) curves.

### Statistical Analysis

Descriptive statistics were reported for continuous variables as means with SDs or medians with IQRs, and frequencies with percentages for categorical variables. For the outcome of mortality at 1 year, we first determined time to event with Kaplan-Meier estimation for each subphenotype. We then fitted Cox proportional hazards models to ascertain hazard ratios and 95% CIs for each subphenotype compared to reference. For reference categories, we used all other subphenotypes compared to each selected subphenotype. These models were adjusted using 21 total features: beneficiary demographics (age, sex, race), beneficiary-level cardiac procedures (implantable cardioverter defibrillator, cardiac catheterization), hospital academic status, hospital number of beds, hospital travel distance, complete area-level SDOH factors, and NCHS urban/rural status (Table S2 in Multimedia Appendix 2). Features were selected for the models based on prior OHCA literature and those that more closely aligned with our outcome of survival at 1 year [2,3,11]. To account for the inclusion of multiple predictor variables, we used a linear predictor as an offset in these models. Statistical analyses were performed using R and Python (vesrion 3.9.3). This study was completed in accordance with the STROBE (Strengthening the Reporting of Observational Studies in Epidemiology) guidelines [27].

### Ethical Considerations

The study was reviewed and approved by the Institutional Review Board at the Icahn School of Medicine at Mount Sinai (21-00976).

## Results

### Overall Cohort

After excluding beneficiaries with missing data, we identified 27,028 unique individuals who survived to discharge after OHCA. Overall, the cohort was 40.1% (n=10,831) female; 15% (n=4055) identified as Black, 79.2% (n=21,407) as White, and 5.8% (n=1566) as “other” beneficiaries (Table 1). For age, 15.4% (n=4156) of the cohort included beneficiaries older than 85 years. Among area-level SDOH, the median HHI by zip code was US $49,720.50 (IQR US $39,893.25-$64,233.25), and the median percentage living below the poverty level at the zip code level was 10.4% (IQR 6.0%-16.4%). Overall mortality at 1 year was 45.1% (n=12,191).

**Table 1.** Overall characteristics by subphenotype.

Features			Subphenotype 1 (n=4417)	Subphenotype 2 (n=9165)	Subphenotype 3 (n=9580)	Subphenotype 4 (n=3866)	Total (n=27,028)	*P* value
**Beneficiary-level demographics**								
	**Sex, n (%)**							
		Male	2080 (47.1)	5577 (60.9)	6057 (63.2)	2483 (64.2)	16,197 (59.9)	<.001
		Female	2337 (52.9)	3588 (39.1)	3523 (36.8)	1383 (35.8)	10,831 (40.1)	
	**Age category (years), n (%)**							
		65–74	2310 (52.3)	4414 (48.2)	4810 (50.2)	2173 (56.2)	13,707 (50.7)	<.001
		75–84	1344 (30.4)	3169 (34.5)	3321 (34.7)	1331 (34.4)	9165 (33.9)	
		≥85	763 (17.3)	1582 (17.3)	1449 (15.1)	362 (9.4)	4156 (15.4)	
	**Race, n (%)**							
		Black	2631 (59.6)	448 (4.9)	504 (5.3)	472 (12.2)	4055 (15)	<.001
		White	1327 (30)	8234 (89.8)	8729 (91.1)	3117 (80.6)	21,407 (79.2)	
		Other	459 (10.4)	483 (5.3)	347 (3.6)	277 (7.2)	1566 (5.8)	
	Elixhauser Comorbidity Index, median (IQR)		14.000 (4.000–23.000)	13.000 (4.000–23.000)	14.000 (4.000–24.000)	14.000 (4.000–24.000)	13.000 (4.000–23.000)	<.001
**Beneficiary-level hospital procedures and dispositions**								
	Cardiac catheterization at index hospitalization, n (%)		843 (19.1)	1767 (19.3)	2937 (30.7)	1342 (34.7)	6889 (25.5)	<.001
	Implantable cardioverter defibrillator placement at index hospitalization, n (%)		351 (7.9)	653 (7.1)	1192 (12.4)	347 (9)	2543 (9.4)	<.001
	Interhospital transfer at index hospitalization, n (%)		107 (2.4)	6 (0.1)	11 (0.1)	3849 (99.6)	3973 (14.7)	<.001
	From skilled nursing facility prior to index OHCA^a^ hospitalization, n (%)		559 (12.7)	531 (5.8)	828 (8.6)	226 (5.8)	2144 (7.9)	<.001
	Inpatient hospital stay prior to index OHCA, n (%)		114 (2.6)	120 (1.3)	285 (3)	155 (4)	674 (2.5)	<.001
	Discharged to skilled nursing facility after index OHCA, n (%)		879 (19.9)	981 (10.7)	1594 (16.6)	1412 (36.5)	4866 (18)	<.001
	Total hospital length of stay in days, median (IQR)		5.000 (1.000–12.000)	5.000 (1.000–12.000)	5.000 (1.000–12.000)	5.000 (1.000–12.000)	5.000 (1.000–12.000)	<.001
	Total number of interhospital transfers mean (SD)		0.024 (0.156)	0.001 (0.026)	0.001 (0.034)	1.135 (0.362)	0.167 (0.424)	<.001
	**Travel distance to index OHCA hospital from residence (kilometers), n (%)**							
		<8.0	2566 (58.1)	6049 (66)	73 (0.8)	1510 (39.1)	10,198 (37.7)	<.001
		8.0-16.1	1182 (26.8)	2639 (28.8)	1328 (13.9)	851 (22)	6000 (22.2)	<.001
		>16.1	669 (15.1)	477 (5.2)	8179 (85.4)	1505 (38.9)	10,830 (40.1)	<.001
**Hospital-level characteristics, n (%)**								
	Minor academic teaching		1406 (31.8)	471 (5.1)	1541 (16.1)	431 (11.1)	3849 (14.2)	<.001
	Major academic teaching		3011 (68.2)	8694 (94.9)	8039 (83.9)	3435 (88.9)	23,179 (85.8)	<.001
	Total number of beds^b^, median (IQR)		7.000 (5.000–8.000)	5.000 (4.000–6.000)	6.000 (4.000–8.000)	5.000 (4.000–7.000)	5.000 (4.000–7.000)	<.001
**Area-level social determinants of health**								
	Household income (US $) at zip code level, median (IQR)		$36,462 ($29,648-$45,309)	$54,375 ($44,320-$72,394)	$50,818 ($41,825.50-$63,549.25)	$51,659.50 ($41,295-$67,081)	$49,720.50 ($39,893.25-$64,233.25)	<.001
	Percentage unemployed at zip code level, median (IQR)		8.4 (6.8–10.4)	5 (3.9–6.2)	5.15 (4–6.6)	5.5 (4.2–7.1)	5.5 (4.2–7.2)	<.001
	Percentage below poverty level at zip code level, median (IQR)		22.1 (16.5–29)	8.4 (4.9–12.8)	9.2 (5.5–14)	9.8 (5.7–15.7)	10.4 (6–16.4)	<.001
	Percentage high school education or higher at zip code level, median (IQR)		79 (72.3–84)	90.5 (85.9–93.9)	88.5 (83.2–92.6)	88.4 (81.8–92.9)	88 (81.6–92.6)	<.001
	Percentage bachelor’s degree or higher at zip code level, median (IQR)		16.9 (11.6–24.6)	28.3 (19.2–43)	21 (14.7–31.3)	24.2 (16.3–36.4)	22.8 (15.5–34.7)	<.001
	Percentage who drive alone at zip code level, median (IQR)		72.9 (58.5–79)	81 (76.8–84.3)	82.3 (78.5–85.6)	80.6 (75.8–84.4)	80.6 (75.4–84.4)	<.001
	NCHS^c^ large metropolitan urban classification, n (%)		3223 (73)	4776 (52.1)	3373 (35.2)	2095 (54.2)	13,467 (49.8)	<.001
	NCHS small/mid metropolitan classification, n (%)		1028 (23.2)	2599 (28.4)	3561 (37.2)	1143 (29.6)	8331 (30.8)	<.001
	NCHS nonmetropolitan classification, n (%)		166 (3.8)	1790 (19.5)	2646 (27.6)	628 (16.2)	5230 (19.4)	<.001
**Outcomes**								
	1-year mortality		2375 (53.8)	4577 (49.9)	4032 (42.1)	1207 (31.2)	12,191 (45.1)	<.001

a OHCA: out-of-hospital cardiac arrest.

b Number of beds: 1=6-24 beds, 2=25-49 beds, 3=50-99 beds, 4=100-199 beds, 5=200-299 beds, 6=300-399 beds, 7=400-499 beds, 8=500 or more beds.

c NCHS: National Center for Health Statistics.

### Characteristics of Subphenotypes by K-Means

**Figure 1.**
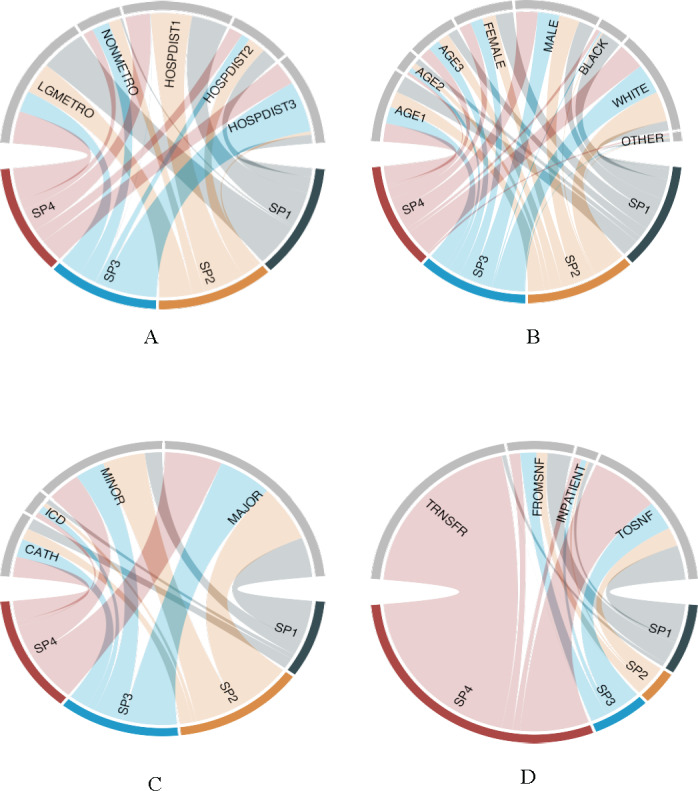
Chord diagrams demonstrating grouped characteristics for each of 4 out-of-hospital cardiac arrest (OHCA) subphenotypes. The diagrams demonstrate the grouped characteristics by each subphenotype. Each chord diagram includes key grouped features and their relationship with each subphenotype. The size of each chord (or arc) is representative of the proportional relationship between each feature and subphenotype. (A) Urban/nonurban and driving distance by subphenotype. (B) Beneficiary-level demographics by subphenotype. (C) Hospital characteristics and procedures by subphenotype. (D) Beneficiary disposition by location for each subphenotype. AGE1: beneficiary level—age category 65-74 years; AGE2: beneficiary level—age category 75-84 years; AGE3: beneficiary level—age category >85 years; BLACK: beneficiary level—Black race; CATH: beneficiary level—cardiac catheterization at index hospitalization; DRIVE: area level—percentage who drive alone at zip code level; ELX: beneficiary level—Elixhauser comorbidity index; FEMALE: beneficiary level—female sex; FROMSNF: beneficiary level—from skilled nursing facility prior to index OHCA; HOSPBEDS: hospital level—total number of beds; HOSPDIST1: beneficiary level—distance to travel to hospital from residence <8.0 kilometers; HOSPDIST2: beneficiary level—distance to travel to hospital from residence 8.0-16.1 kilometers; HOSPDIST3: beneficiary level—distance to travel to hospital from residence >16.1 kilometers; ICD: beneficiary level—implantable cardioverter defibrillator placement at index hospitalization; INPATIENT: beneficiary level—inpatient hospital stay at index OHCA; LGMETRO: area level—National Center for Health Statistics large metropolitan urban classification; LOS: beneficiary level—total hospital length of stay in days; MAJOR: hospital level—major academic teaching; MINOR: hospital level—minor academic teaching; NONMETRO: area level—National Center for Health Statistics nonmetro classification; OTHER: other beneficiary race/ethnicity (Centers for Medicare and Medicaid Services–defined categories Hispanic, Asian, Native Hawaiian or Pacific Islander, American Indian, or Alaska Native); SP1: subphenotype 1; SP2: subphenotype 2; SP3: subphenotype 3; SP4: subphenotype 4; TOSNF: beneficiary level—to skilled nursing facility after index OHCA; TRNSFR: beneficiary level—interhospital transfer at index hospitalization; TRSNFRTOT: beneficiary level—total number of interhospital transfers; WHITE: beneficiary level—White race.

**Figure 2.**
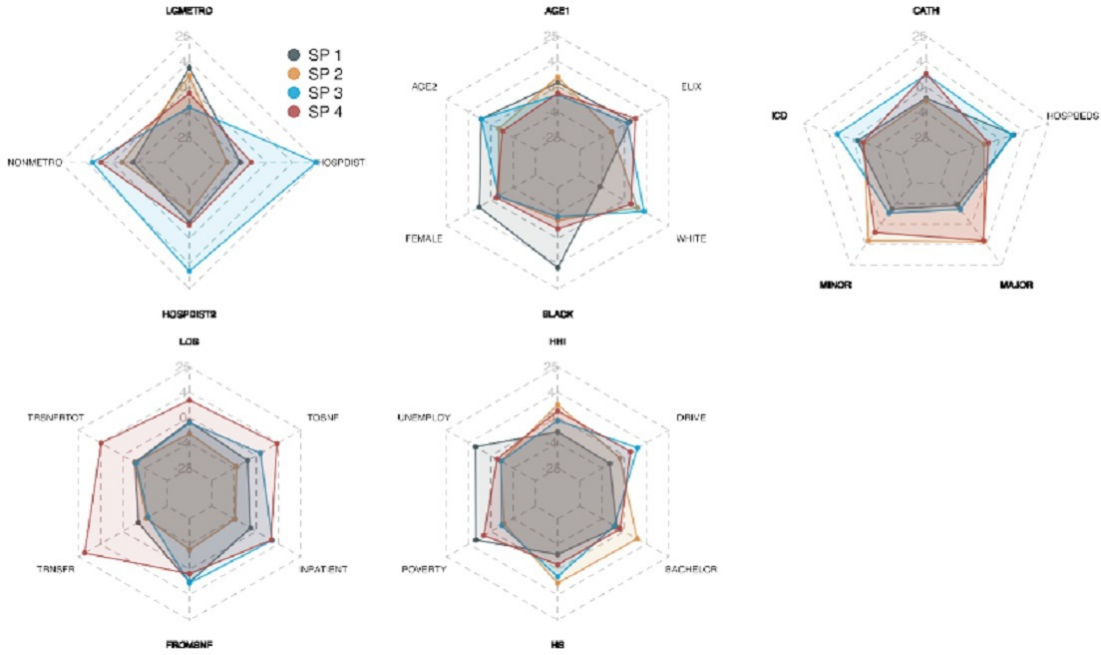
Radar plots demonstrating the degree of association between normalized features and cluster membership. The radar plots represent the degree of association between normalized features and cluster membership. Each point represents a coefficient from the multinomial regression model for subtypes using normalized features (note that we do not normalize binary features), and each grid line represents −25, −4, 0, 4, and 25, respectively, where axes are scaled with squared root. As an example, a 1 unit increase in normalized household income (HHI) increases log odds of subtype 1-4 by −2.0, 1.6, −0.1, and 0.4. AGE1: beneficiary level—age category 65-74 years; AGE2: beneficiary level—age category 75-84 years; BLACK: beneficiary level—Black race ; CATH: beneficiary level—cardiac catheterization at index hospitalization; DRIVE: area level—percentage who drive alone at zip code level; ELX: beneficiary level—Elixhauser comorbidity index; FEMALE: beneficiary level—female sex; FROMSNF: beneficiary level—from skilled nursing facility prior to index OHCA; HOSPBEDS: hospital level—total number of beds; HOSPDIST1: beneficiary level—distance to travel to hospital from residence <8.0 km; HOSPDIST2: beneficiary level—distance to travel to hospital from residence 8-16 kilometers; ICD: beneficiary level—implantable cardioverter defibrillator placement at index hospitalization; INPATIENT: beneficiary level—inpatient hospital stay at index OHCA; LGMETRO: area level—National Center for Health Statistics large metropolitan urban classification; LOS: beneficiary level—total hospital length of stay in days; MAJOR: hospital level—major academic teaching; MINOR: hospital level—minor academic teaching; NONMETRO: area level—National Center for Health Statistics nonmetro classification; SP1: subphenotype 1; SP2: subphenotype 2; SP3: subphenotype 3; SP4: subphenotype 4.; TOSNF: beneficiary level—to skilled nursing facility after index OHCA; TRNSFR: beneficiary level—interhospital transfer at index hospitalization; TRSNFRTOT: beneficiary level—total number of interhospital transfers; WHITE: beneficiary level—White race.

We identified 4 distinct subphenotypes that were statistically and significantly different based on distributions of features. Distributions can be seen in the chord diagrams in Figure 1, and the relationship of normalized features and cluster membership is shown in Figure 2.

### Subphenotype 1

Subphenotype 1 (n=4417) included the largest distribution of female and Black beneficiaries, 52.9% (n=2337) and 59.6% (n=2631) respectively, as well as Other benefciaries at 10.4% (n=459), who resided in more NCHS urban-classified zip codes (n=3323, 73%). A greater proportion were transferred from a SNF prior to index hospitalization for OHCA. Compared to other subphenotypes, beneficiaries in this group had the lowest rates of cardiac catheterization at index hospitalization, at 19.1% (n=843). Subphenotype 1 had, notably, several of the least robust area-level SDOH measures: the lowest median HHI at US $36,462 (IQR US $29,648-$45,309), highest unemployment at 8.4% (IQR 6.8%-10.4%), and highest percentage living below the poverty level at 22.1% (IQR 16.5%-29%). For outcomes, this subphenotype had the highest 1-year mortality at 53.8% (n=2375).

### Subphenotype 2

Subphenotype 2 (n=9165) was characterized by a greater distribution of White and male beneficiaries, the smallest distribution of Black beneficaries, and had the strongest zip code–level SDOH measures. This included the highest median HHI at US $54,375 (IQR US $44,320-$72,394), highest median percentage high school education or higher at 90.5% (IQR 85.9%-93.9%), highest median bachelor’s degree or higher at 28.3% (IQR 19.2%-43%), and the lowest median unemployment at 5% (IQR 3.9%-6.2%). For subphenotype 2, 1-year mortality was 49.9%(n=4577).

### Subphenotype 3

This subphenotype (n=9580) included the largest demographic representation of White beneficiaries and had the highest rate of cardiac catheterization at 34.7% (n=1342), the greatest distribution with a driving distance to index OHCA hospital from primary residence >16.1 kilometers at 85.4% (n=8179), and the highest rate of discharge to a SNF after index hospitalization at 36.5% (n=1412) compared to the other subphenotypes. One year mortality was 41.2% (n=4032).

### Subphenotype 4

Subphenotype 4 (n=3866) was characterized by the greatest distribution of the beneficiaries undergoing interhospital transfer at index hospitalization at 99.6% (n=3849) and included a large distribution of male (n=2483) and White (n=3117) beneficiaries. Among zip code–level SDOH measures, beneficiaries in subphenotype 4 had moderate median HHI at US $51,659.50 (IQR US $41,295-$67,081) and moderate to high median percentage unemployed at 5.5% (IQR 4.2%-7.1%) compared to other subphenotypes. This subphenotype had the lowest 1-year mortality at 31.2% (n=1207).

### Association of Subphenotypes and Primary Outcomes

**Figure 3.**
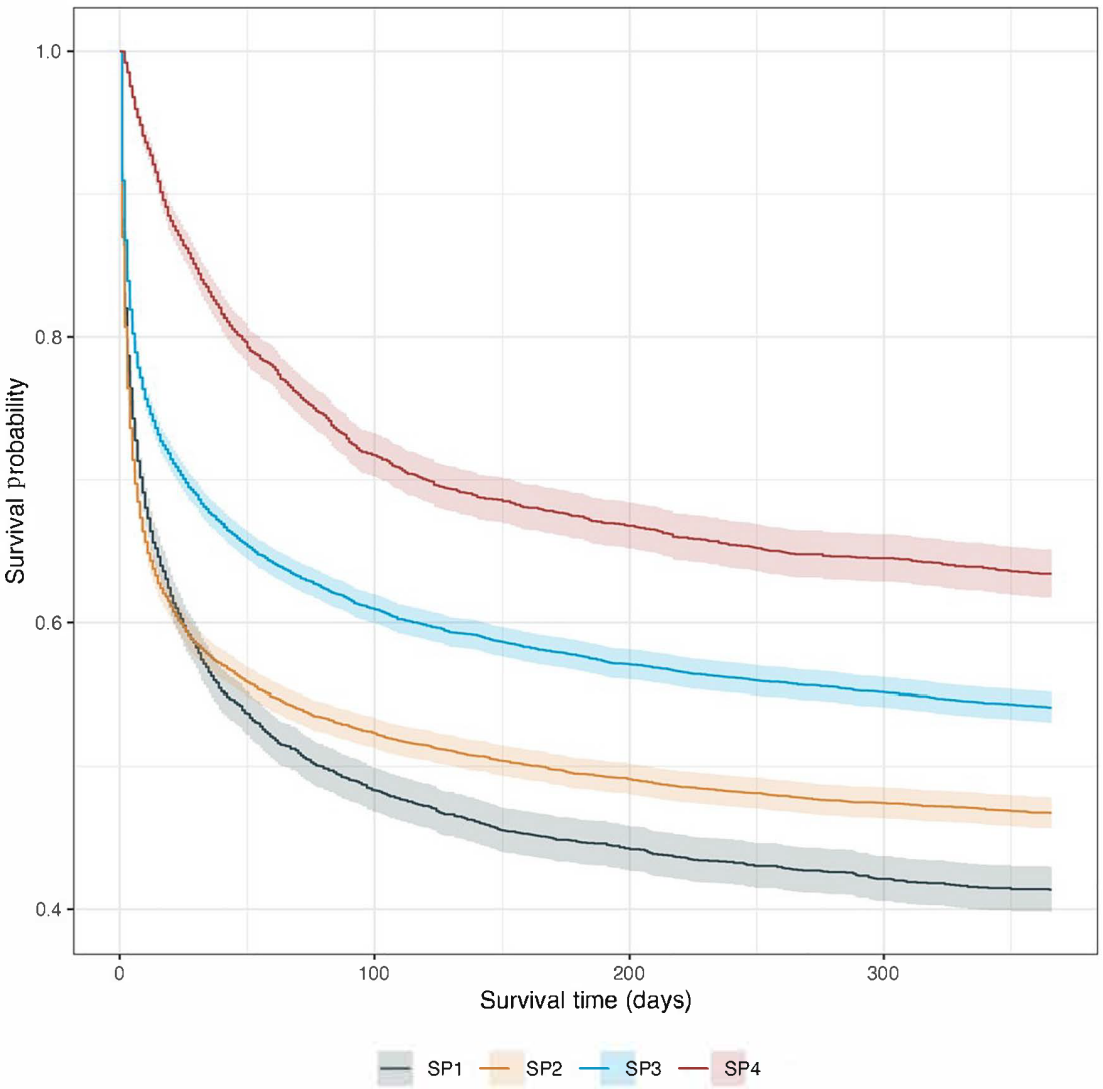
Survival by Kaplan Meier estimation for 1-year mortality for each of the 4 out-of-hospital cardiac arrest subphenotypes. SP1: subphenotype 1; SP2: subphenoytype 2; SP3: subphenotype 3; SP4: subphenotype 4.

**Figure 4.**
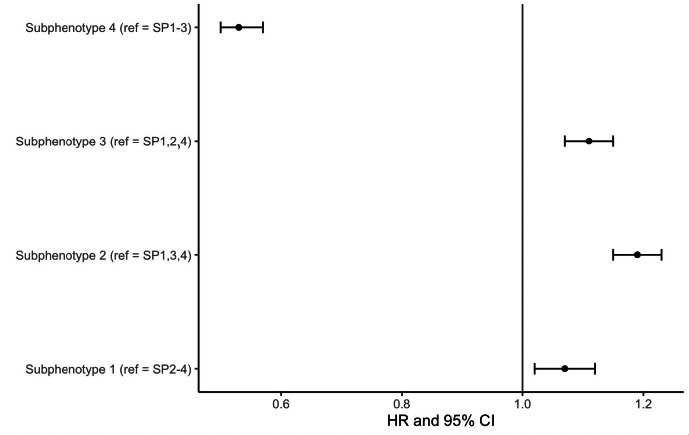
Forest plot of Cox proportional hazards models for each subphenotype for outcome of mortality at 1 year using a linear predictor as an offset. Models are adjusted for 21 total features, including beneficiary demographics (age, sex, race), beneficiary-level cardiac procedures (implantable cardioverter defibrillator, cardiac catheterization), hospital academic status, hospital number of beds, hospital travel distance, complete area-level social determinant of health factors, and National Center for Health Statistics urban/rural status (Table S2 in Multimedia Appendix 4). Each subphenotype model is compared to reference (ie, all other subphenotypes). HR: hazard ratio.

One year survival by Kaplan Meier estimation is shown in Figure 3 and Table 2. Subphenotype 1 demonstrated the steepest mortality, with a median survival of 80 days (95% CI 64-99 days) and subphenotype 4 had the highest probability of survival at one year. In fully adjusted models evaluating the primary outcome of mortality at 1 year, subphenotype 4 had a decreased hazard of death at 1 year (hazard ratio [HR] 0.53, 95% CI 0.50-0.57) compared to reference (all other subphenotypes) (Figure 4). For all other subphenotypes (1-3) we found an increased hazard of death compared to reference (subphenotype 1: HR 1.07, 95% CI 1.02-1.12; subphenotype 2: HR 1.19, 95% CI 1.15-1.23; subphenotype 3: HR 1.11, 95% CI 1.07-1.15).

**Table 2.** Time to event for Kaplan-Meier estimation.

	At risk, n	Events, n	Days, mean	Median (95% upper, lower confidence limit)
Subphenotype 1	4417	2375	173.3	80 (64, 99)
Subphenotype 2	9165	4577	187.9	131 (131, 207)
Subphenotype 3	9580	4032	218.4	N/A^a^
Subphenotype 4	3866	1207	257.8	N/A

a N/A: not applicable.

## Discussion

### Principal Findings

In this unsupervised machine learning cluster analysis, we identified 4 unique and distinct OHCA subphenotypes among Medicare beneficiaries using multi-modal data. The characteristics of these subphenotypes are distinguished by both beneficiary demographics and area-level SDOH such as zip code–level HHI, poverty, education, and unemployment. For subphenotype 1, we found high 1-year mortality was tied to poor area-level SDOH factors and subphenotype 4 was tied with moderate SDOH factors and lowest unadjusted 1-year mortality. After complete adjustment and joint modeling of these features, we noted an increased hazard of death for subphenotypes 1 to 3 but not for subphenotype 4 when compared to reference (ie, all other subphenotypes). This exploratory work provides further insight into the complex interaction of nonclinical factors in health outcomes and has identified potential methodological approaches for other patient populations or data sets.

Research using machine learning or clustering approaches and incorporating SDOH factors for predictive modeling of OHCA outcomes is limited, with most prior work using individual-level clinical or prehospital features for outcome prediction, and none using Medicare data [28-31]. Of note, one recent study used the city of Chicago Cardiac Arrest Registry to Enhance Survival (CARES) data merged with multi-modal community-level data to evaluate if social and environmental factors can increase predictive accuracy of models. The authors found, compared to base models using registry data alone, that model accuracy was significantly improved when including important community and social determinants to predict neurological outcomes [32]. A prior study of OHCA patients with nonshockable rhythms using a machine learning latent class approach identified 4 clinically distinct subphenotypes associated with neurological and mortality outcomes at 30 days, finding that arterial partial pressure of oxygen, patient age, and serum potassium had the highest discriminatory power; however, this study did not examine area- or individual-level SDOH. Several studies have also found contrasting results, with area-level SDOH factors not demonstrating strong associations with outcomes. In a non-OHCA study specifically assessing the predictive performance of neighborhood-level SDOH for risk prediction, the authors found that SDOH data did not improve models beyond baseline electronic health record data [33].

Our work has identified 4 unique Medicare beneficiary subphenotypes tied to long-term OHCA outcomes in the context of several SDOH domains. Descriptively, we identified important characteristics among our subphenotypes, including differences in distributions across race, sex, key hospital cardiac procedures, rates of interhospital transfer, and zip code–level SDOH factors, such as poverty, HHI, and unemployment. Overall, in models that included adjustment for SDOH, clinical, and demographic factors, the hazard of death at 1 year persisted and was increased across subphenotypes 1 to 3 but decreased for subphenotype 4. This mortality risk was notably highest among subphenotypes 2 and 3 compared to other subphenotypes. This suggests that certain SDOH domains may not modify mortality risk and clinical and demographic factors are drivers of differences in survival. The decreased risk of morality at 1 year for subphenotype 4 was also potentially modified by more robust SDOH factors, but likely represents attributes unique to this subphenotype. The majority of subphenotype 4 underwent interhospital transfer at index OHCA. This could have potentially incurred a survival benefit due to escalation of care at the receiving hospital. Additionally, subphenotype 4 had the smallest distribution of patients aged older than 85 years and high rates of implantable cardioverter defibrillator and cardiac catheterization, potentially leading to differences in outcomes. This could reflect the high morbidity and mortality in OHCA at the extremes of age, as well as improved survival and clinical outcomes for select patients undergoing cardiac catheterization or implantable cardioverter defibrillator placement at index hospitalization.

The results of this study have implications for future work, which could explore if our identified subphenotypes are associated with other OHCA outcomes such as readmission and health care expenditures, as well as their place in the context of broader SDOH domains. This approach could serve to better identify groups of beneficiaries who are at risk for worse postdischarge trajectories after OHCA. Further work is needed to elucidate our findings and examine actionable and modifiable social factors tied to OHCA survival. However, we believe our proposed approach is scalable and feasible and could be applied to emergency care conditions and health outcomes in the context of SDOH factors.

### Limitations

There are several limitations to this work that should be noted. Because we are using claims and not cardiac arrest registry data, identification of the cohort may lack similar sensitivity and specificity for OHCA. This could result in potential misclassification of OHCA cases. Additionally, using zip codes as our geographic unit of analysis as opposed to smaller areas, such as census tract or neighborhood level, and using individual-level SDOH data may have limited our ability to identify a robust association with SDOH and clinical outcomes after OHCA. Overall, some of our results could be potentially attributable to the SDOH domains we selected for this study. These domains were not comprehensive and did not include other important SDOH (food insecurity, housing insecurity). Also, it is important to note that the racial composition of Medicare data included more than 75% of beneficiaries who identified as White and 10% of beneficiaries who identified as Black in 2013 [34]. This limits our ability to closely examine outcomes across a robust population that includes representative races and ethnicities for the United States. Despite these limitations, this exploratory research has identified important subphenotypes of beneficiaries linked to SDOH factors who may be at risk for poor long-term outcomes. These areas could be targets for improved in-hospital care or discharge planning to improve long-term survival.

### Conclusions

In this machine learning cluster analysis examining the association of area-level SDOH factors with long-term outcomes for a cohort of Medicare beneficiaries who experienced an OHCA, we identified 4 distinct clusters and important associations with SDOH measures and mortality at 1 year. After adjustment, we found an increased hazard of death at 1 year for subphenotypes 1 to 3 and decreased hazard for subphenotype 4 when compared to reference (all other subphenotypes). These results suggest that area-level SDOH measures may be associated with OHCA outcomes, but further work is needed to determine if other individual- or area-level SDOH domains are more closely tied to long-term survival.

## Supplementary material

10.2196/51844 Multimedia Appendix 1 Table S1. Complete list of proposed features.

10.2196/51844 Multimedia Appendix 2 Uniform manifold approximation and projection of k-means clustering (panel 1) vs hierarchical clustering (panel 2).

10.2196/51844 Multimedia Appendix 3 Comparison of subphenotypes using k-means and hierarchical clustering algorithms. KM: k-means; HC: hierarchical clustering; SP: subphenotype.

10.2196/51844 Multimedia Appendix 4 Features included for cox proportional hazard regression models by subphenotype.

## Abbreviations

CARES: Cardiac Arrest Registry to Enhance Survival
CMS: Centers for Medicare and Medicaid Services
ED: emergency department
HHI: household income
HR: hazard ratio
*ICD-9-CM*: *International Classification of Diseases, Ninth Revision, Clinical Modification*
LOS: length of stay
MedPAR: Medicare Provider Analysis and Review
NCHS: National Center for Health Statistics
OHCA: out-of-hospital cardiac arrest
OSRM: Open-Source Routing Machine
RIF: research identifiable file
ROC: receiver operating characteristic
SDOH: social determinants of health
SNF: skilled nursing facility
STROBE: Strengthening the Reporting of Observational Studies in Epidemiology
UMAP: uniform manifold approximation and projection
