# Supplementary material for: Joint Modeling of Social Determinants and Clinical Factors to Define Subphenotypes in Out-of-Hospital Cardiac Arrest Survival: Cluster Analysis
Source: JMIR Aging. 2023 Dec 6;6:e51844. doi: 10.2196/51844 (PMC10721134; doi:10.2196/51844)
Supplement: Multimedia Appendix 1 [file aging-v6-e51844-s001.docx]

| **Feature** | **Description** | **Abbreviation** |
| --- | --- | --- |
| Age category 65-74 y. | Beneficiary Level: Age Category 65-74 years old | AGE1 |
| Age Category: 75-84 y. | Beneficiary Level: Age Category 65-74 years old | AGE2 |
| Age category 85+ y. | Beneficiary Level: Age Category >85 years old | AGE3 |
| Sex: Female | Beneficiary Level: Sex Female | FEMALE |
| Race: Black | Beneficiary Level Race: Black | BLACK |
| Race: White | Beneficiary Level Race: White | WHITE |
| Race and Ethnicity: Other | Beneficiary Race/Ethnicity: Other (CMS defined categories Hispanic; Asian/Native Hawaiian, or Pacific Islander; American Indian or Alaska Native) | OTHER |
| Elixhauser Comorbidity Index | Beneficiary Level: Elixhauser Comorbidity Index | ELIX |
| Cardiac Catheterization at Index Hospitalization | Beneficiary Level: (Y) Cardiac Catheterization at Index Hospitalization | CATH |
| ICD Placement at Index Hospitalization | Beneficiary Level: (Y) ICD Placement at Index Hospitalization | ICD |
| Interhospital Transfer | Beneficiary Level: (Y) Interhospital Transfer at Index Hospitalization | TRNSFR |
| From Skilled Nursing Facility Prior to Index OHCA Hospitalization | Beneficiary Level: From Skilled Nursing Facility Prior to Index OHCA | FROMSNF |
| Inpatient hospital Stay Prior to Index OHCA | Beneficiary Level: Inpatient Hospital Stay Index OHCA | INPATIENT |
| Transfer to SNF after Index OHCA | Beneficiary Level: To Skilled Nursing Facility After Index OHCA | TOSNF |
| Total Length of Stay (LOS) for Index OHCA | Beneficiary Level: Total Hospital Length of Stay in Days | LOS |
| Total Number of Interhospital Transfers | Beneficiary Level: Total Number of Interhospital Transfers | TRSNFRTOT |
| Hospital Travel Distance | Beneficiary Level: Distance to travel to hospital from residence | HOSPDIST1=<5 miles HOSPDIST2= 5-10 miles HOSPDIST3 = >10 miles |
| Hospital Academic Affiliation: Minor Academic | Hospital Level Characteristics: Minor Academic Teaching | MINOR |
| Hospital Academic Affiliation: Major Academic | Hospital Level Characteristics: Major Academic Teaching | MAJOR |
| Total Number of Hospital Beds | Hospital Level: Total Number of Beds | HOSPBEDS |
| Median Household Income | Area Level: median household income ZIP code level | HHI |
| Percent Unemployed | Area Level: Percent Unemployed ZIP code level | UNEMPLOY |
| Percent Below the Poverty Line | Area Level: Percent Below Poverty level ZIP code level | POVERTY |
| Percent High School Education or Higher | Area Level: Percent High School Education or Higher ZIP code level | HS |
| Percent bachelor’s degree or Higher | Area Level: Percent bachelor’s degree or Higher ZIP code level | BACHELOR |
| Percent Drive Alone | Area Level: Percent Drive Alone ZIP code | DRIVE |
| National Center for Health Statistics (NCHS) Large Metropolitan Classification | Area Level: NCHS Large Metropolitan Urban Classification | LGMETRO |
| National Center for Health Statistics (NCHS) Non- Metro Classification | Area Level: NCHS Non-Metro Classification | NONMETRO |
