# Supplementary figures and images for: Joint Modeling of Social Determinants and Clinical Factors to Define Subphenotypes in Out-of-Hospital Cardiac Arrest Survival: Cluster Analysis
Source: JMIR Aging. 2023 Dec 6;6:e51844. doi: 10.2196/51844 (PMC10721134; doi:10.2196/51844)

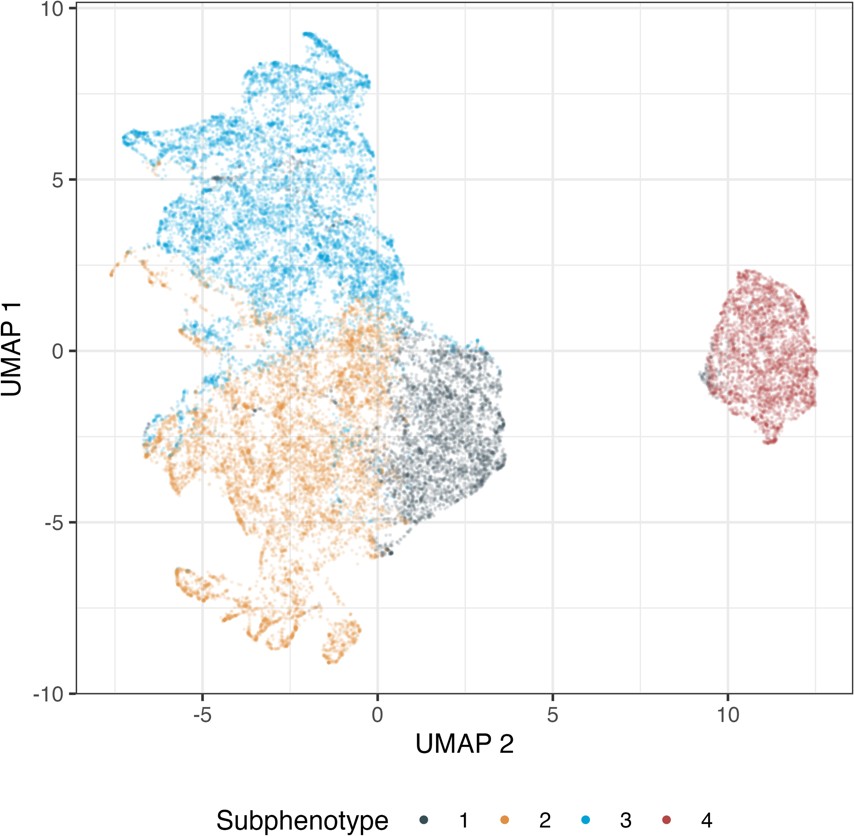

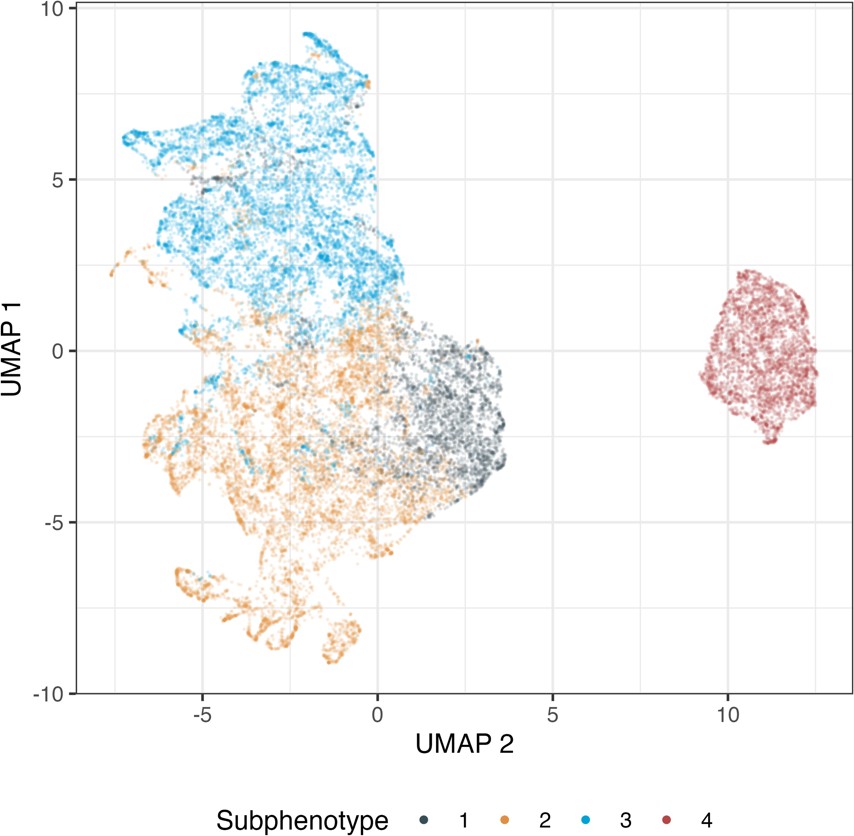

Supplement: Multimedia Appendix 2 [file aging-v6-e51844-s002.docx]

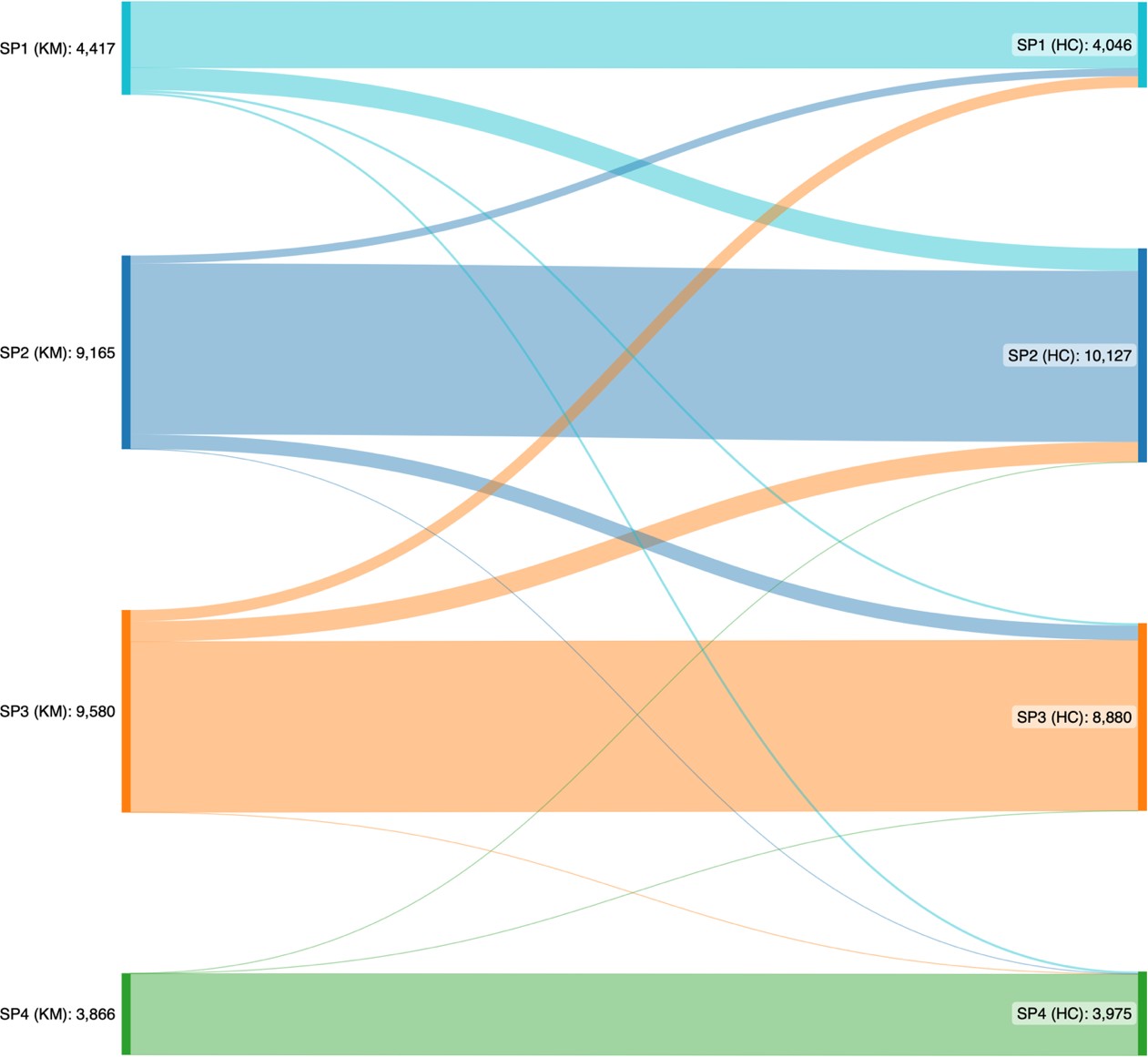

Supplement: Multimedia Appendix 3 [file aging-v6-e51844-s003.docx]
