# Supplementary material for: Joint Modeling of Social Determinants and Clinical Factors to Define Subphenotypes in Out-of-Hospital Cardiac Arrest Survival: Cluster Analysis
Source: JMIR Aging. 2023 Dec 6;6:e51844. doi: 10.2196/51844 (PMC10721134; doi:10.2196/51844)
Supplement: Multimedia Appendix 4 [file aging-v6-e51844-s004.docx]

| Features |
| --- |
| Beneficiary Level: Demographics (age + sex + race) |
| Beneficiary Level: Elixhauser Comorbidity Index |
| Beneficiary Level: (Y/N) Cardiac Catheterization at Index Hospitalization |
| Beneficiary Level: (Y/N) ICD Placement at Index Hospitalization |
| Beneficiary Level: Distance to travel to hospital 5-10 |
| Beneficiary Level: Distance to travel to hospital >10 |
| Hospital Level Characteristics: Minor Academic Teaching |
| Hospital Level Characteristics: Major Academic Teaching |
| Hospital Level: Total Number of Beds (<100, 1000-399, >400) |
| Area Level: NCHS Large Metropolitan Urban Classification |
| Area Level: NCHS Non-Metro Classification |
| Area Level: median household income ZIP code level |
| Area Level: Percent Unemployed ZIP code level |
| Area Level: Percent Below Poverty level ZIP code level |
| Area Level: Percent High School Education or Higher ZIP code level |
| Area Level: Percent bachelor’s degree or higher ZIP code level |
| Area Level: Percent Drive Alone ZIP code |
